# Disparities in Long-Acting Reversible Contraceptive Utilization among Married Women in Ethiopia: Findings of the Ethiopian Demographic and Health Survey

**DOI:** 10.1155/2020/3430975

**Published:** 2020-03-05

**Authors:** Biniyam Tadesse Haile, Yohannes Ejigu Tsehay

**Affiliations:** ^1^Jimma University, Institute of Health Sciences, Department of Health Economics, Management and Policy, Jimma, Ethiopia; ^2^International Centre for Health Monitoring and Evaluation, Jimma University, Jimma, Ethiopia

## Abstract

**Background:**

Long-acting contraceptive methods, subdermal implants, and intrauterine devices are reliable, safe, and cost-effective family planning methods. However, these methods are not widely used in Ethiopia despite government effort to increase access. The study is aimed at assessing the rate of utilization of long-acting contraceptive methods among married women and associated factors.

**Method:**

We analyzed the 2016 Ethiopian Demographic and Health Survey dataset. A total of 2045 married women of reproductive age group, who were using any modern contraceptive method at the time of the survey, were included in the study. Descriptive statistics were computed to characterize the study participants. Bivariate and multivariable logistic regression analyses were performed to identify associated factors, reporting odds ratios (ORs) and 95% confidence intervals (95% CIs).

**Result:**

The multivariable analyses showed that women educational status, parity, religion, previous history of abortion, desire for more child, and region where the respondents reside were significantly the factors that determine the utilization of long-acting contraceptive. There is a significant regional disparity in long-acting contraceptive utilization. Compared to women residing in Tigray region, those who live in other regions (Afar-Somali, Oromia, Amhara, Benishangul Gumz-Gambela, and Southern Nations Nationalities and People) have low likelihood of using long-acting contraceptive methods.

**Conclusion:**

Utilization of long-acting family planning method is low in Ethiopia. There is a significant regional disparity in utilizing these methods. Policy makers should promote culture-sensitive and tailored interventions to improve the utilization of long-acting family planning methods.

## 1. Introduction

Access to reproductive health, particularly family planning, is a human right [1, 2]. Ensuring universal access to family planning has positive impact on gender equality, women's empowerment, and ultimately reduction of poverty [1–3]. Globally, contraceptive prevalence rate has almost doubled, from 35% in 1970 to 63% in 2017. However, the contraceptive prevalence rate in Africa showed a gradual increase from 8% to 36% over the same period [1].

Women and children are given a particular attention in the health policy of Ethiopia. The policy recommends decentralizing services and enriching the concept and intensifying the practice of family planning for optimal family health and planned population dynamics. Accordingly, the ministry is engaged in a range of efforts to improve access and quality of modern family planning services. As a result, the contraceptive prevalence rate has increased by more than fourfold during the last fifteen years, from 8% in 2000 to 36% in 2016 [4].

On the other hand, the proportion of women currently using long-acting contraceptive (LAC) remained significantly lower than short-acting methods [4–7]. Long-acting contraceptive (LAC) methods including subdermal implants and intrauterine devices (IUDs) are believed to be reliable, safe, and cost-effective family planning methods. Unlike short-acting methods, such as pills, condom, and injectables, LAC methods require less frequent health facility visit, and their effectiveness does not depend on user's adherence [1, 2].

Various studies conducted in Ethiopia showed that utilization of long-acting contraceptives is influenced by social, cultural, economic, and health system-related factors. Particularly, women's social status, access to education, and their involvement in decision making on reproductive matters, social norms regarding marriage, childbearing, and sexuality were found to be the major determinants of long-acting contraceptive use [5–9]. Furthermore, the capacity of health systems to provide quality and accessible family planning services greatly affects utilization of long-acting contraceptive methods [2, 10]. However, these studies were conducted in specific geographic areas and had limited power to generalize the findings to the entire country.

This study is therefore aimed at assessing the rate of long-acting contraceptive method utilization among married women using modern contraceptives in Ethiopia and identifying the associated factors.

## 2. Methods and Materials

We followed the methods of Ejigu and Tadesse [11]. We used the data from the EDHS 2016 survey, which was conducted between January and June 2016. The survey covered all the nine regions and two city administrations of Ethiopia. It was designed to be a representative of the country and each region by taking a sampling frame from the 2007 population census of Ethiopia. The current study focuses on analyzing disparity in utilization of long-acting contraceptive methods among married women.

### 2.1. Sampling Strategy and Data Collection Field Work

The survey employed a stratified, two-stage cluster sampling technique. Ethiopia has nine administrative regions and two city administrations. The regions are divided into zones, districts, and kebeles (the lowest administrative units). A total of 84,915 census enumeration areas (EAs) were created by dividing kebeles with an average of 181 households per EA. Samples of 645 EAs (202 from urban and 443 from rural areas) were randomly selected, and 28 households from each EA were included in the survey by applying a systematic random sampling strategy. The survey administered questionnaires and collected biological sample from women of reproductive age group (15 to 49 years), children under five years old, and men aged between 15 and 59 years. However, women of the reproductive age group were the source population, and married women of the reproductive age group were the study population of this particular study. We used the data collected from women of reproductive age group (15–49 years) using DHS's women's questionnaire. The questionnaire was a standardized and field-tested tool used in different countries, and only a subset of variables were included in the current study. Data quality was maintained through pretesting of the data collection tools prior to the survey, and data were collected by qualified and trained survey teams, in which each team consisted of one supervisor, one field editor, four female interviewers, and two male interviewers. In addition, independent quality assurance team was deployed to check the quality of the collected data through random spot checking at all sites of the survey.

### 2.2. Outcome Variable

The outcome variable is the utilization of long-acting contraceptive (LAC) methods. Utilization of LAC is defined as the use of implant/implanol or intrauterine devices (IUCD) at the time of survey. Hence, women who are currently using LAC methods are categorized as users (Yes), and those who are using modern methods other than LACs were grouped under nonusers (No).

### 2.3. Predictor Variables

The study included potential predictors that could determine utilization of modern contraceptives. Sociodemographic characteristics (age, educational status, religion, employment status, etc.), wealth index/category (poor, middle, and rich), place of residence (urban/rural), region of residence, obstetric history (number of children, history of abortion, mode and place of delivery for the recent birth, etc.), main decision maker on contraceptive use (husband, the women, and joint decision making), and current fertility preference were predictor variables.

### 2.4. Data Analysis

Descriptive statistics were computed to summarize the sociodemographic characteristics of study participants. Moreover, bivariate logistic regression analyses were conducted to determine the presence of association between dependent and independent variables. Multivariable logistic regression analysis was performed to measure the independent effect of each independent variables on long-acting contraceptive method utilization. The multivariate logistic regression analysis was conducted by using a stepwise backward elimination technique after including all relevant covariates in the model and eliminating one variable at a time. Variables which with *p* values < 0.05 were considered significant. Data analysis was carried out by Statistical Package for Social Science (SPSS) version 20.

### 2.5. Ethical Issues

The study was approved by the Federal Democratic Republic of Ethiopia Ministry of Science and Technology and the Institutional Review Board of ICF International. Data collectors were trained for one month on different aspects of the data collection process, including ethical issues. Moreover, verbal consent was secured for each respondent prior to interview, and involvement of participants was based on their willingness, voluntary.

## 3. Result

A total of 2045 married women of the reproductive age group, who are currently using the modern contraceptive method, were included in this study. Majority of the respondents 67.9% were rural dwellers, 52.4% were Orthodox Christianity religion followers, 46.1% had no formal education, and 43.5% of respondents were unemployed. Regarding wealth of the household, 46%, 24.4%, and 29.5% were found under rich, middle, and poor category, respectively (Table 1).

More than half of the women had 3 or less children, where vast majority of them (71.2%) had pregnancy within the past five years, prior to the survey. More than one-fourth of recently born children were due to unwanted pregnancy, while 9.2% of women had history of abortion in their lifetime. Regarding fertility preference, 60.2% of the study participants want to have another child; out of which, 35.4% want to have a child within less than 3 years. About half of the study participants had similar desire for child their respective partners/husbands, whereas 20.6% of women had more desire than their partner/husband (Table 2).

Only 3 in ten modern family planning users use long-acting contraceptive methods, where implants/Norplant is used by 80.1% of long-acting contraceptive method users. Injectable/Depo-Provera is a predominantly used method, which accounts 62.2% of all types of modern contraceptive methods. Regarding decision making to use of contraceptive method, 73.6% of women made joint decision with their partner, 21.1% of respondents made the decision alone, and 5.1% husbands/partners made decision alone. (Table 3).

### 3.1. Determinants of Long-Acting Reversible Contraceptive Use

The multivariable analyses showed that women educational status, parity, religion, previous history of abortion, desire for more child, and region where the respondents reside were significantly the factors that determine the utilization of long-acting contraceptive.

Compared to women with no formal education, those who attended up to primary level where 24% less likely to use LAC methods (AOR = 0.76, at 95% CI: 0.62, 0.94), whereas women with higher-level educational status were more likely to use LAC (AOR = 1.42, at 95% CI: 1.01, 2.02). Women who have less than three children are less likely to use LAC compared to those with 3 or more children (AOR = 0.28, at 95% CI: 0.14, 0.56). The study showed a significant disparity in LAC utilization across administrative regions. Compared to women residing in Tigray region, those who live in other regions (Afar-Somali, Oromiya, Amhara, Benishangul Gumz-Gambela, and SNNP) have low likelihood of using long-acting contraceptive. On the other hand, household wealth and place of residence (urban/rural) were not significant predicators of LAC utilization (Table 4).

## 4. Discussion

This study is mainly aimed at describing regional disparity in LAC utilization among married women and also identifying sociodemographic and economic determinants. The current study revealed that utilization of LAC methods among married women using modern family planning methods was low. Only 30.1% of women were using LAC methods. Different factors such as educational status, parity, religion, previous history of abortion, women desire for more child, and region were independent predictors of long-acting contraceptive use.

Our finding is fairly comparable with other studies conducted in various parts of the country [6, 9, 12], whereas the result is higher than similar study done in Bangladesh (18.0%) and Iran (21.4%) [12, 13]. The discrepancy could be due to the difference in sociodemographic, cultural, and economic characteristics of study participants and health system of those countries in general.

Increasing utilization of LAC method has positive socioeconomic impact on users, their families, and on the society as a whole [14, 15]. Due to their long-term effect, users of LAC will have much less frequent visit to health facility than short-acting contraceptive users, where higher frequency of visit is linked with financial and social implications due to travel cost and time. Moreover, the effectiveness of LACs does not depend on user's adherence and regular availability of drugs or supplies. Hence, these methods are more reliable, safe, and cost-effective than short-term contraceptives [14–16]. Cognizant of these benefits, the government of Ethiopia has been promoting long-acting family planning methods; however, our study and previous studies indicated that its successes were mixed. In our study, we found unacceptable level of regional disparity in LAC utilization where Harari-Diredawa-Addis Ababa had higher proportion (44.5%) of LAC users, followed by Tigray (34.8%), but Afar-Somali had the lowest proportion (13.0%) of LAC users. The finding also indicates wrong targeting; Afar-Somali regions are predominantly pastoralist, with mobile population; and these populations should have been prioritized over others because in these, population may not have access to health service all the time due to their mobility and LACs could have greatly reduced the need for health facility visits to collect family planning methods. Though this study does not assess regional variation in health care delivery system, our finding of regional disparity can be used as a proxy indicator for the existence of inequitable distribution of health services among the different regional states. Further qualitative study is recommended to explore implementation of family planning programs across regions, equity in health care services, and social and cultural factors which could result in regional disparity in long-acting contraceptive method utilization.

Various literatures have shown that urban residents have better socioeconomic status (literacy, wealth, and access to information) which have positive contribution to access and utilization of contraceptive methods [5, 6]. In contrary, the current study showed that LAC utilization is not affected by women's place of residence and household wealth. Our finding is consistent with the results of similar study conducted in Bangladesh [13]. Lack of significant effect of the place of residence on the use of LAC method could be due to strong effort made by the government and nongovernment actors in improving primary health service coverage in rural areas to ensure equity through its universal primary health coverage initiative. In addition, the implementation of community health programs (rural health extension program) bridges the gap between the community and health facilities and ensures access to health service in rural areas. Moreover, the finding could be due to the fact that most modern family planning methods including LAC are provided by public health facilities free of out-of-pocket payment.

In this study, compared to women with no formal education, those with primary education had decreased likelihood LAC use. This finding is not consistent with the findings of similar studies conducted in Gondar [6], Western Ethiopia [17], Tigray [8], West Arsi Zone [12], and Afghanistan [18], where utilization of LAC progressively increases with women's level of education. In most cases, women's education is linked with improved health literacy and informed choice and decision making. However, the finding has only borderline significance and could be due to a chance or unmeasured confounding.

Compared to women with three or more children, those who had no child were less likely to use LAC methods. Our finding is also consistent with similar studies conducted in Mekele [5], Arbaminch Zuriya district [9], and Bale Zone [19], where LAC utilization increases with parity/number of children. This can be explained by the fact that women with more children want to limit their families and they tend to prefer permanent and long-acting contraceptives, due to its comparative advantage in spacing childbirth.

The study used a nationally representative survey data, and the findings could be generalizable to the whole country. However, the study should be understood in light of the following limitations. Due to the nature of cross-sectional survey, it is difficult to establish cause-effect relationship. Moreover, the supply side factors are important determinants of long-acting contraceptive utilization. However, in this study, the influence of only demand side factors was evaluated.

## 5. Conclusion

Utilization of long-acting contraceptive methods remained low in Ethiopia. There is a significant disparity in the utilization of long-acting contraceptives among women living in different regions of the country. Policy makers from government and nongovernment organizations should design culture-sensitive and tailored interventions to promote utilization of long-acting contraceptive methods. Moreover, government and nongovernmental organizations working in provision of family planning should target rural areas and pastoralist areas to ensure equitable access to modern family planning service in general and long-acting family planning methods in particular. A national study to evaluate the supply side factors including service access and quality-related factors influencing long-acting contraceptive use among Ethiopian women is warranted.

## Figures and Tables

**Table 1:** Sociodemographic and economic characteristics of study participants.

Characteristics	Frequency (*n*)	Percent (%)
Age of the respondent (years)		
15-24	519	25.4
25-34	949	46.4
34 and above	577	28.2
Religion		
Orthodox	1071	52.4
Protestant	465	22.7
Muslin	485	23.7
Others	24	1.2
Educational level		
No formal education	942	46.1
Primary	719	35.2
Secondary	228	11.1
Higher	156	7.6
Occupation		
Unemployed (not working)	889	43.5
Working	1156	56.5
Husband/partner's education level		
No education	655	32.0
Primary	848	41.5
Secondary	285	13.9
Higher	257	12.6
Husband/partner's occupation		
Unemployed (not working)	87	4.3
Working	1958	95.7
Household wealth of category		
Poor	603	29.5
Middle	500	24.4
Rich	942	46.1
Place of residence		
Urban	656	32.1
Rural	1389	67.9
Region (province)		
Tigray	210	10.3
Afar and Somali	54	2.6
Amhara	432	21.1
Oromia	289	14.1
Benishangul and Gambela	300	14.7
Southern Nations, Nationalities and People (SNNP)	367	17.9
Harari, Diredewa, and Addis Ababa	393	19.2

**Table 2:** Obstetric history and fertility preferences of study participants.

Characteristics	Frequency (*n*)	Percent (%)
Parity		
None	145	7.1
1 child	1102	53.9
2 children	561	27.4
≥3 children	237	11.6
Births in last five years		
No	589	28.8
Yes	1456	71.2
Ever terminated pregnancy		
No	1856	90.8
Yes	189	9.2
Wanted status of last child (*n* = 1456)		
Wanted	1084	74.5
Unwanted	372	25.5
Place of birth of most recent child (*n* = 956)		
Hospital, health facility	584	60.5
Other	381	39.5
Last birth a caesarean section (*n* = 1456)		
No	1379	94.7
Yes	77	5.3
Ever been tested for HIV		
No	652	31.9
Yes	1393	68.1
Current respondent desire for more children		
Want no more	814	39.8
Want more	1231	60.2
Preferred waiting time to have another child (*n* = 1231)		
≤3 years	724	35.4
>3 years	465	22.7
Undecided/unknown	42	2.1

**Table 3:** Frequency distribution of modern contraceptive use and source of method among married in Ethiopia.

*N* = 2045	Frequency (*n*)	Percent (%)
Category of currently used contraceptive method		
Long-acting	617	30.2
Short-acting	1428	69.8
Currently used contraceptive method by type		
Injectable (Depo-Provera)	1272	62.2
Pill	146	7.1
Implants/Norplant	494	24.2
IUD	123	6.0
Condoms	10	0.5
Source of currently used contraceptive method		
Government health facility	1642	80.3
Other sources (pharmacy, private clinics, and shops)	403	19.7
Decision maker for using contraception		
Joint decision making	1509	73.8
Mainly respondent (women)	432	21.1
Mainly husband/partner	104	5.1

**Table 4:** Determinants of LARC use among married women in Ethiopia.

Characteristics/variables (*n* = 2045)	Use of LAC method		Unadjusted OR (95%CI)	Adjusted OR (95%CI)
	No	Yes		
Age of the women (years)				
15-24	391	128	1.00	1.00
25-34	635	314	1.51 (1.20, 1.92)^∗^	1.26 (0.95, 1.68)
34 and above	402	175	1.33 (1.02, 1.74)^∗^	1. 00 (0.67, 1.48)
Highest educational level				
No education	644	298	1.00	1.00
Primary	531	188	0.71 (0.44, 1.14)	0.73 (0.57, 0.94)^∗^
Secondary	159	69	0.79 (0.40, 1.56)	0.71 (0.48, 1.04)
Higher	94	62	1.32 (0.59, 2.94)	1.03 (0.66, 1.60)
Religion				
Orthodox	725	346	1.00	1.00
Protestant	329	136	0.76 (0.62, 0.94)^∗^	1.46 (1.05, 2.02)^∗^
Muslin	356	129	0.94 (0.69, 1.28)	0.71 (0.48, 1.04)
Other^∗∗^	18	6	1.42 (1.01, 2.02)^∗^	1.21 (0.46, 3.21)
Parity				
No child	113	32	0.59 (0.36, 0.94)^∗^	0.46 (0.24, 0.86)^∗^
1 child	755	347	0.95 (0.71, 1.30)	0.75 (0.50, 1.14)
2 children	400	161	0.84 (0.60, 1.16)	0.72 (0.50, 1.03)
≥3 children	160	77	1.00	1.00
Wealth category				
Poor	438	165	0.85 (0.67, 1.06)	0.97 (0.75, 1.24)
Middle	338	162	1.08 (0.85, 1.36)	1.08 (0.85, 1.34)
Rich	652	290	1.00	1.00
Region				
Tigray	137	73	1.00	1.00
Afar-Somali	47	7	0.28 (0.12, 0.65)^∗^	0.30 (0.12, 0.73)^∗^
Amhara	290	142	0.92 (0.65, 1.30)	0.91 (0.64, 1.31)
Oromia	215	74	0.65 (0.44, 0.95)^∗^	0.56 (0.37, 0.86)^∗^
Benishangul Gumz-Gambela	243	57	0.44 (0.29, 0.66)^∗^	0.41 (0.27, 0.64)^∗^
SNNP	278	89	0.60 (0.42, 0.87)^∗^	0.42 (0.26, 0.70)^∗^
Harari-Diredawa-Addis Ababa	218	175	1.51 (1.06, 2.13)^∗^	1.60 (1.04, 2.42)^∗^
Place of residence				
Urban	425	231	1.41 (1.16, 1.72)^∗^	1.04 (0.76, 1.43)
Rural	1003	386	1.00	1.00
Previous history of abortion				
No	1284	572	1.43 (1.01, 2.02)^∗^	1.72 (1.19, 2.47)^∗^
Yes	144	45	1.00	1.00
Desire for more children				
Wants no more	564	250	1.00	1.00
Wants more	864	367	0.96 (0.79, 1.15)	1.72 (1.20, 2.47)^∗^

^∗^Statistically significant with *p* value < 0.05; other ^∗∗^*Wakefeta* (traditional) and Catholics.

## Data Availability

The data used to support the findings of this study are available in SPSS and STATA data sets at the website of Demographic and Health Survey. Interested individuals or groups can access the data by requesting the responsible body using the following link: https://dhsprogram.com/data/
